# A Novel Machine Learning Model for Predicting Natural Conception Using Non-Laboratory-Based Data

**DOI:** 10.1007/s43032-025-01927-2

**Published:** 2025-07-14

**Authors:** Yeliz Kaya, Yunus Aydın, Coşkun Kaya, Tuğba Tahta, Özer Çelik

**Affiliations:** 1 Department of Gynecology and Obstetrics Nursing, Eskişehir Osmangazi University Faculty of Health Sciences, Eskişehir, Türkiye; 2 Denizli Saglik Hospital IVF Center, Denizli, Türkiye; 3 Department of Urology, Health Science University Eskisehir City HARH, Eskişehir, Türkiye; 4 Health Services Vocational School, Ankara Medipol University, Ankara, Türkiye; 5 Department of Mathematics and Computer, Eskişehir Osmangazi University, Eskisehir, Türkiye

**Keywords:** Natural conception, Machine learning, Fertility prediction, Sociodemographic factors, Couple-based analysis

## Abstract

This study aimed to predict the likelihood of natural conception among couples by using a machine learning (ML) approach based on sociodemographic and sexual health data. This marks a novel, non-invasive methodology for fertility prediction. This prospective study included 197 couples divided into two groups: 98 fertile couples (Group 1) who achieved natural conception within one year, and 99 infertile couples (Group 2) who were unable to conceive despite regular unprotected intercourse. Data were collected using a structured form capturing 63 variables from both partners. Using the Permutation Feature Importance method, 25 key predictors were selected. The variables included BMI, age, menstrual cycle characteristics, and varicocele presence. Five ML models were developed and their performance was evaluated using metrics such as accuracy, sensitivity, and specificity. The XGB Classifier showed the highest performance among the models tested with an accuracy of 62.5% and a ROC-AUC of 0.580, indicating limited predictive capacity. The selected predictors encompassed a balance of medical, lifestyle, and reproductive factors for both partners, emphasizing the couple-based approach. Key factors included BMI, caffeine consumption, history of endometriosis, and exposure to chemical agents or heat. This study assessed the use of ML to predict natural conception using sociodemographic and health data The key predictors identified emphasize the importance of couple-based and lifestyle factors in predicting natural conception. However, the predictive capacity of the models was limited, highlighting the need for future studies with larger datasets and expanded predictors to improve accuracy and facilitate AI integration into fertility assessment.

**Supplementary Information:**

The online version contains supplementary material available at 10.1007/s43032-025-01927-2.

## Introduction

Natural conception is of paramount importance for couples, serving not only as an indicator of reproductive capability but also as a reflection of overall reproductive health. Accurately estimating the likelihood of natural conception is essential to aid couples in making informed decisions regarding their reproductive plans, whether they are actively attempting to conceive or seeking their fertility potential. Predicting natural conception remains a challenging endeavor due to the multifactorial nature of conception influenced by biological, sociodemographic, behavioral, and environmental factors. To address this complexity, predictive models have been developed to estimate the probability of natural conception [1–3]. However, these models have predominantly relied on traditional statistical methods. While these approaches have provided valuable insights, they are often limited in their ability to account for the nonlinear and dynamic interactions among the various factors influencing conception. Moreover, such models have yet to establish a concrete role in clinical practice, making them unreachable for healthcare providers and couples seeking guidance.

The growing application of artificial intelligence in fertility research has introduced a novel dimension to this field, offering the potential for more comprehensive and precise predictive models [5–7]. Among these, artificial intelligence has emerged as a promising tool for addressing the limitations of traditional approaches. However, to date, only one study has utilized ML learning to differentiate fertile and infertile couples, employing anthropometric, antioxidative, and metabolic signatures [8].

In this study, we aimed to predict whether couples are likely to conceive a child after one year of regular, unprotected intercourse using basic sociodemographic characteristics and sexual health history data through machine learning (ML). This novel approach marks the first attempt to utilize such data for predicting reproductive outcomes, offering a simplified yet effective model that does not require extensive clinical or biochemical testing.

## Materials and Methods

### Data Source and Study Workflow

This prospective study was conducted following ethical approval obtained from the relevant ethics committee. Data were collected from two distinct clinical settings to establish the study groups. Group 1 (fertile couples) couples who attended the pediatric outpatient clinic for the evaluation of their children and met the inclusion criteria of achieving natural conception within one year of regular, unprotected intercourse. Group 2 (infertile couples) was derived from patients seeking treatment at the IVF Center of Denizli Private Health Hospital meeting the criteria of being unable to conceive after 12 months of regular, unproteprograms, and diagnostic tools cted intercourse.

### Data Preprocessing

The study included two distinct groups:

- **Group 1 (Fertile Couples):** This group comprised couples in which the female partner successfully conceived spontaneously within one year of engaging in regular, unprotected sexual intercourse. Additionally, the pregnancy resulted in the live birth of a child who, at the time of data collection, was under the age of two. Importantly, none of the couples in this group had used any form of ART to achieve conception. The inclusion criteria ensured that these couples represented those with proven natural fertility within a typical timeframe.
- **Group 2 (Infertile Couples):** This group consisted of couples who had been unable to conceive after 12 consecutive months of regular, unprotected sexual intercourse. Unlike previous studies that focused solely on idiopathic infertility, this group included all couples who failed to achieve conception, regardless of whether the cause of infertility was known or unknown. No additional clinical or diagnostic criteria were used to exclude couples with potential medical or unexplored reproductive issues, providing a broad representation of non-conceiving couples.

To establish these groups, the following inclusion and exclusion criteria were meticulously determined:

### Inclusion Criteria

1. Participants aged 18 or older.
2. Voluntary consent to participate in the study.
3. **For Group 1 (Fertile Couples):** Couples in which the female partner achieved spontaneous conception within one year of regular, unprotected intercourse, and had a child who was under two years of age at the time of the clinic visit.
4. **For Group 2 (Infertile Couples):** Couples unable to conceive despite 12 months of regular, unprotected intercourse.
5. Couples reporting a frequency of sexual intercourse of at least twice a week during the last year.

### Exclusion Criteria

1. Participants younger than 18.
2. Refusal to provide consent for participation.
3. **For Group 1 (Fertile Couples):** Couples unable to achieve spontaneous conception or having a child who was under two years of age at the time of the clinic visit.
4. **For Group 2 (Infertile Couples):** Couples with secondary infertility or known medical conditions that preclude natural conception (e.g., vasectomy, anorchia, anovulation).
5. Previous history of pregnancy, miscarriage, or curettage in either group.
6. Any medical or surgical condition known to adversely affect fertility (e.g., endometriosis, severe male factor infertility).
7. Couples with a history of assisted reproductive technology (ART) use.
8. Known hormonal imbalances or genetic conditions affecting fertility.
9. For male partners: having any known male-factor infertility, such as azoospermia or severe oligospermia.

#### Data Collection

The data were collected using a structured data collection form designed by the researchers based on a review of the relevant literature. The form included the sociodemographic characteristics and sexual health history of the participants A total of 33 parameters were collected for female partners and 30 parameters for male partners. These variables are categorized under the following headings:

#### Female Partner

- ○ Sociodemographic data: Age, height, weight.
- ○ Lifestyle factors: Smoking, alcohol consumption, caffeine intake, exercise habits.
- ○ Medical history: Depression, chronic medication use, systemic illnesses, history of thromboembolism, cancer or genetic conditions, and history of chemotherapy or radiotherapy.
- ○ Reproductive history: Menstrual cycle regularity, duration, age at menarche, and previous pregnancy outcomes (e.g., live births, miscarriages, ectopic pregnancies).
- ○ Gynecological history: History of polycystic ovary syndrome, uterine surgeries, endometriosis, hormonal imbalances, and congenital uterine abnormalities.

#### Male Partner

- ○ Sociodemographic data: Age, height, weight.
- ○ Lifestyle factors: Smoking, alcohol consumption, caffeine intake, exposure to heat.
- ○ Medical history: Chronic conditions, cancer or genetic conditions, and history of chemotherapy or radiotherapy.
- ○ Reproductive history: Cryptorchidism, varicocele, testicular trauma, and erectile or ejaculatory dysfunction.
- ○ Fertility-related factors: Puberty onset age, presence of secondary sexual characteristics, and any history of infertility or subfertility.

#### ML Models

In this study, several ML models were employed to classify couples based on their likelihood of achieving natural conception. The models included the Random Forest Classifier, Light Gradient Boosting Machine (LGBM) Classifier, Extra Trees Classifier, Extreme Gradient Boosting (XGB) Classifier, and Logistic Regression algorithms, each offering unique strengths and capabilities for classification tasks.

The Random Forest Classifier is an ensemble learning method that constructs multiple decision trees during training and outputs the mode of the classes for classification. It is particularly effective for datasets with a mix of numerical and categorical features as it requires minimal preprocessing and is robust against overfitting. Furthermore, its ability to measure feature importance makes it a valuable tool for identifying the most influential variables in predicting fertility outcomes. Similarly, the LGBM Classifier, known for its speed and efficiency, is well-suited to large datasets and complex patterns, efficiently capturing feature interactions and delivering scalable performance. The Extra Trees Classifier shares similarities with Random Forest but introduces additional randomness in selecting split points for decision trees. This added randomness often enhances generalization and stability, particularly in noisy datasets or when dealing with variables that have overlapping distributions. The XGB Classifier, on the other hand, builds on the traditional gradient boosting methods by incorporating advanced regularization techniques, making it highly effective in high-dimensional datasets and robust against overfitting. Finally, Logistic Regression, as a simple linear model, provides a baseline comparison. While it may not capture complex nonlinear relationships as effectively as tree-based methods, it offers clear interpretability and computational efficiency, making it suitable for identifying linear associations between variables.

Each of these models brings distinct advantages to the characteristics of the variables used in this study, which include a combination of sociodemographic and health-related data. By employing these diverse algorithms, the study ensures a comprehensive evaluation of model performance, ultimately looking to identify the most effective approach to predict the likelihood of natural conception accurately. The ML algorithms were carried out using Python software (Python Software Foundation. Python Language Reference, version 3.5. Available at http://www.python.org).

#### Performance Metrics

In this study, we partitioned the dataset by allocating 80% for model training and 20% for testing purposes. The performance of the models was evaluated using standard classification metrics, including accuracy, sensitivity, specificity, and the area under the receiver operating characteristic (ROC) curve (AUC). These metrics were derived from the confusion matrix, which compares predicted outcomes with actual labels and is commonly used to assess the quality of classification models. Additionally, we applied cross-validation techniques to assess the generalizability and robustness of the models and to facilitate a reliable comparison across different machine-learning algorithms.

#### Feature Selection

The feature selection process was performed by the Permutation Feature Importance method. This evaluates the importance of each variable by individually permuting the values of a feature and measuring the resulting decrease in the model’s prediction performance, qualified by the coefficient of determination (R^2^) score. This method was applied to the complete dataset to identify the most influential factors associated with natural conception.

**Fig. 1:**
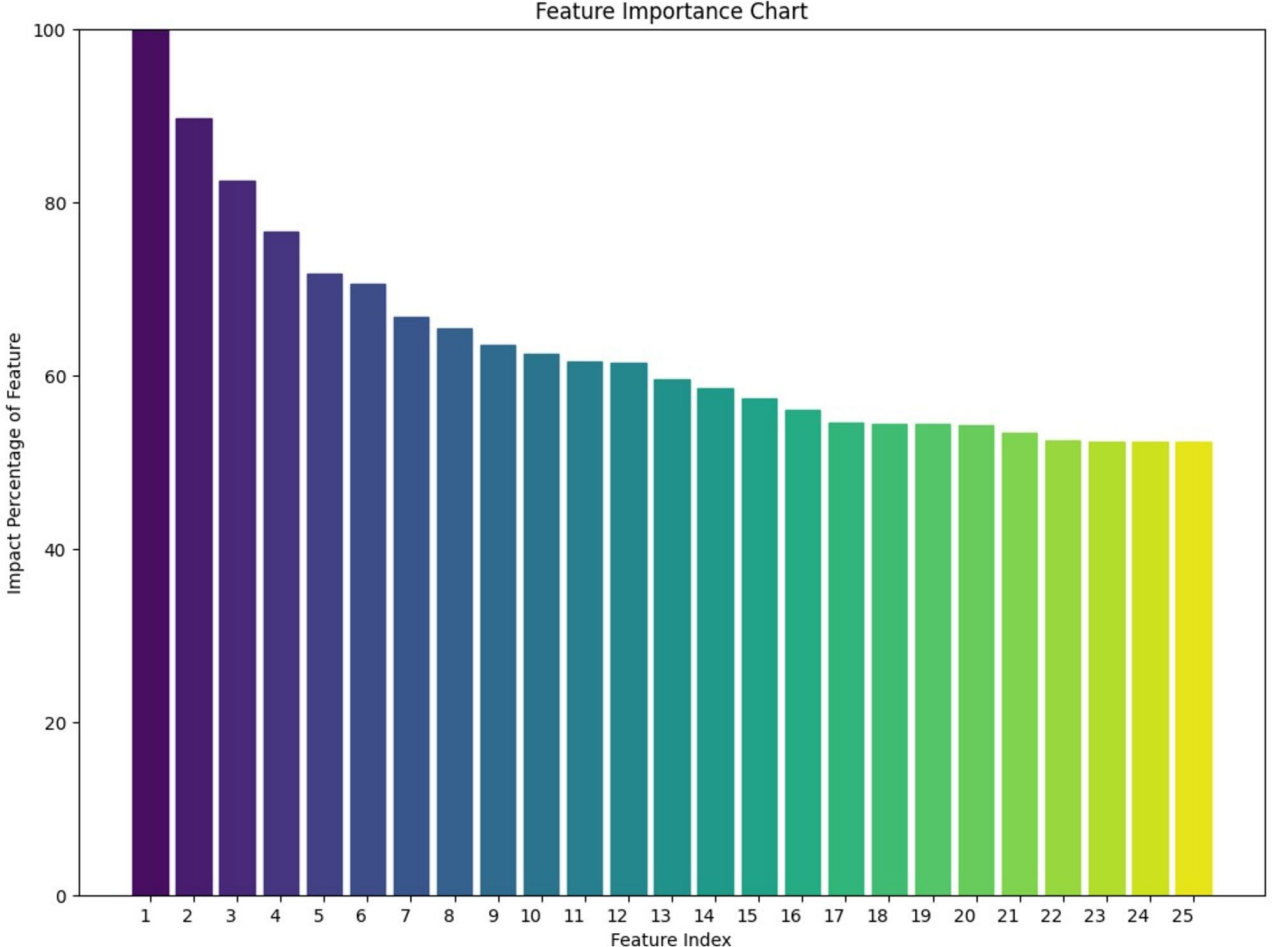
Impact factors of the variables. Female Variables: 1: Body mass index 4: History of family planning use 5: History of vaginismus 6: Age at menarche 7: Presence of hirsutism 8:Age 9: Presence of regular menstruation 10: Duration of menstruation 11:History of cancer 13: History of endometriosis 14: Continuous medication use 17: Caffeine consumption 20: History of uterine surgery 21: Presence of systemic disease 22: Family history of thromboembolism Male Variables: 2: Age at puberty onset 3: Body mass index 15: Regular exercise 16: Continuous medication use 18: Presence of secondary sexual characteristics 19: Presence of varicocele 23: Exposure to chemical agents 24: Exposure to heat 25: Family history of thromboembolism

Out of the 63 parameters collected from the participants, 25 key variables were selected that influence natural conception. These factors include a balanced representation of both female and male partners, representing the couple-based approach of this study, and encompass sociodemographic, lifestyle, medical, and reproductive factors. These variables include body mass index, age, and for females, factors such as age at menarche, the presence of regular menstruation, duration of menstruation, history of family planning use, history of vaginismus, presence of hirsutism, history of uterine surgery, history of endometriosis, and the presence of systemic diseases. For all genders, variables such as a history of cancer, continuous medication use, and a family history of thromboembolism were also significant. Male-specific factors included age at puberty onset, regular exercise habits, the presence of secondary sexual characteristics, varicocele, and exposure to chemical agents or heat. The Permutation Feature Importance Plot is given in Fig. 1.

## Results

To form the study groups, participants were assessed from two distinct sources. For Group 1 (fertile couples), a total of 259 couples were initially evaluated from the Denizli Health Hospital pediatric outpatient clinic. For Group 2 (infertile couples), a total of 312 couples seeking treatment at the IVF Center were assessed. After applying the inclusion and exclusion criteria, 98 couples were included in Group 1, and 99 couples were included in Group 2.

The baseline characteristics of female and male partners were analyzed using data collected through a standardized form. The median age of female partners was 30.0 years (5–95th percentile: 23.0–39.0), with a median body mass index (BMI) of 26.4 (5–95th percentile: 21.6–35.8). Key factors such as age of menarche (median: 13.0 years), age of puberty onset (median: 13.0 years), and menstrual duration (median: 5.0 days) were also recorded. Among female partners, notable lifestyle and medical history factors included 41.1% reporting daily caffeine use, 24.4% reporting continuous medication use, and 21.3% having a history of polycystic ovary syndrome (PCOS). Regular menstruation was present in 87.8%, while 6.6% had a history of vaginismus. Uterine surgeries and endometriosis were reported in 17.3% and 5.6%, respectively (Table 1).

**Table 1:** Summary of baseline characteristics of female partners*

	Median	5–95th percentile
Age	30.0	23.0—39.0
BMI	26.4	21.6—35.8
	**Present (n. %)**	**Absent (n. %)**
Smoking	12 (6.1)	185 (93.9)
Daily caffeine use	81 (41.1)	116 (58.9)
Depression history	19 (9.6)	178 (90.4)
Endometriosis history	11 (5.6)	186 (94.4)
Hormonal problem history	26 (13.2)	171 (86.8)
Regular menstruation	173 (87.8)	24 (12.2)
PCOS history	42 (21.3)	155 (78.7)

*BMI* Body Mass Index

^*^ Full descriptive details are provided in Supplementary Table S1

Male partners had a median age of 33.0 years (5–95th percentile: 27.0–43.0) and a median BMI of 26.4 (5–95th percentile: 21.6–35.8). Smoking was prevalent among 52.8%, with 43.1% reporting regular exercise habits. Heat exposure was documented in 8.6%, while 13.2% had a history of varicocele, and 7.1% had undergone varicocelectomy (Table 2). Detailed descriptive statistics of female and male partners are provided in Supplementary Table S1 and Supplementary Table S2.

**Table 2:** Summary of baseline characteristics of male partners*

	Median	5–95th percentile
Age	30.0	23.0—39.0
BMI	26.4	21.6—35.8
	**Present (n. %)**	**Absent (n. %)**
Smoking	104 (52.8)	93 (47.2)
Alcohol consumption	59 (29.9)	138 (70.1)
Daily caffeine use	107 (54.3)	90 (45.7)
Cryptorchidism history	3 (1.5)	194 (98.5)
Varicocele	26 (13.2)	171 (86.8)
Varicocelectomy history	14 (7.1)	183 (92.9)
Erectile dysfunction	3 (1.5)	194 (98.5)
Family planning history	40 (20.3)	157 (79.7)
Family infertility history	5 (2.5)	192 (97.5)

*BMI* Body Mass Index

^*^ Full descriptive details are provided in Supplementary Table S2

The performance of different ML algorithms used to predict the likelihood of achieving natural conception within one year is summarized below in Table 3.

**Table 3:** Prognosis prediction results of different machine learning algorithms

Model Name	ROC-AUC	Accuracy	Precision	Sensitivity	Specificity	Interval
Random forest	0.522	0.525	0.526	0.50	0.55	0.37–0.680
XGB classifier	0.580	0.625	0.632	0.60	0.65	0.475–0.775
Logistic regression	0.432	0.503	0.500	0.50	0.50	0.345–0.655
LGBM	0.473	0.501	0.500	0.75	0.25	0.345–0.655
Extra trees classifier	0.586	0.575	0.579	0.55	0.60	0.422–0.728

*LGBM* Light gradient boosting machine; *XGB* eXtreme gradient boosting, *ROC-AUC* Receiver operating characteristic—area under the curve

Five models, the Random Forest Classifier, LGBM Classifier, Extra Trees Classifier, XGB Classifier, and Logistic Regression, were evaluated using metrics such as ROC-AUC, accuracy, precision, sensitivity, specificity, and also confidence intervals for ROC-AUC. The XGB Classifier achieved the highest accuracy (0.625) and precision (0.632) among the models. Its ROC-AUC was 0.580, sensitivity was 0.60, and specificity was 0.65, with a confidence interval range of 0.475–0.775. While it demonstrated the best performance in the study, its predictive power was found to be limited.

**Fig. 2:**
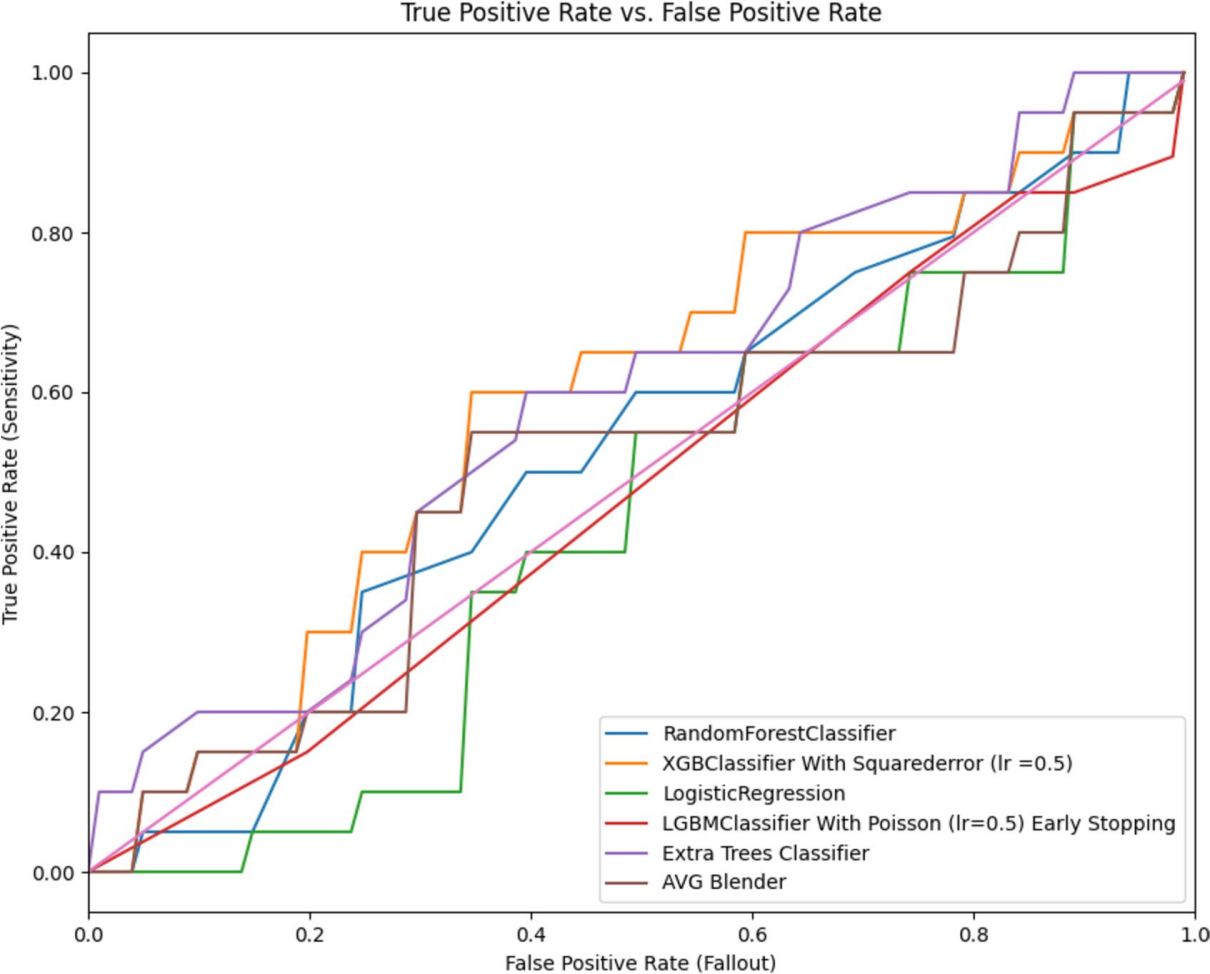
Receiver operating characteristic curve graphs. *LGBM* Light gradient boosting machine; *XGB* eXtreme gradient boosting

The Extra Trees Classifier followed closely with a ROC-AUC of 0.586 and an accuracy of 0.575. Its precision was 0.579, sensitivity was 0.55, and specificity was 0.60, with a confidence interval range of 0.422–0.728. The Random Forest Classifier showed a ROC-AUC of 0.522 and an accuracy of 0.525. Its precision was 0.526, sensitivity was 0.50, and specificity was 0.55. The confidence interval for its ROC-AUC ranged from 0.37 to 0.680. The LGBM Classifier had a ROC-AUC of 0.473 and an accuracy of 0.501, with a precision of 0.500, sensitivity of 0.75, and specificity of 0.25. Its ROC-AUC confidence interval ranged from 0.345 to 0.655. Logistic Regression performed the least effectively, with a ROC-AUC of 0.432 and an accuracy of 0.503. Its precision, sensitivity, and specificity were all 0.500, with a ROC-AUC confidence interval of 0.345–0.655 (Table 3 and Fig. 2).

## Discussion

For partners, conceiving naturally without medical intervention and within a short time-to-pregnancy period holds significant importance. As natural conception is influenced by a complex interplay of biological, behavioral, and lifestyle factors, several studies have explored strategies to enhance its likelihood by focusing on behavioral interventions, educational programs, and diagnostic tools [6–14]. Among these, behaviors such as timing sexual intercourse with the fertility window and adopting optimal sexual positions are often highlighted. However, many misconceptions persist regarding the role of these factors in improving fertility [15]. Recent educational initiatives and studies addressing these misconceptions have aimed to enhance fertility awareness, empowering couples with accurate information to optimize their chances of conception [16–18].

Diagnostic interventions, including hysteroscopy, laparoscopy, and semen analysis, have been employed not only to investigate infertility but also to predict natural conception outcomes [19–21]. These tools provide valuable insights into anatomical and functional factors that may influence fertility. For instance, studies have demonstrated the use of hysteroscopy and laparoscopy in identifying and addressing subclinical conditions that might hinder conception [19, 20]. Similarly, semen analysis serves as a cornerstone for evaluating male fertility and predicting the likelihood of natural conception [21].

Numerous studies have focused on understanding predictive factors and probabilities for natural conception, particularly among couples with unexplained infertility [21–23]. For unexplained infertility, studies show a considerable range of natural conception probabilities, often dependent on factors such as the duration of infertility, female age, and body mass index [21, 22]. For instance, shorter durations of subfertility and younger female age are consistently associated with higher natural conception rates [21, 22]. Conversely, endometriosis and prolonged subfertility are unfavorable predictors [22].

In the literature, a few statistical models have been developed to estimate the likelihood of natural conception among couples. One of the most notable is the Hunault model (2004), which synthesizes data from three studies on subfertile couples and uses predictors such as female age, subfertility duration, primary or secondary infertility, motile sperm percentage, and referral level [1]. Its inclusion of the postcoital test (PCT), a now outdated diagnostic tool, significantly enhances its discriminatory ability. However, the model's reliance on limited predictors and PCT limits its applicability in modern clinical practice. Building on this, the Bensdorp model (2017) incorporated additional predictors, including female BMI, cycle length, basal FSH levels, and semen parameters (volume and morphology), leading to improved calibration and discrimination (c-statistic: 0.71 vs. 0.59 for the Hunault model) [2]. While this model accounts for contemporary clinical challenges, such as rising obesity and delayed childbearing, it still relies on retrospective data and lacks extensive external validation, reducing its generalizability to diverse populations. The dynamic prediction model (2017) by van Eekelen et al. marked a shift from static approaches, providing time-dependent probabilities for natural conception based on evolving clinical data [3]. Although validated externally in a Scottish cohort [4], its reliance on repeated assessments and robust data collection infrastructure makes implementation challenging in routine practice. Despite their contributions, these models have struggled to find widespread clinical use due to several factors, such as the reliance on invasive tests, limited validation, and the complexity of implementation in real-world settings. These factors collectively highlight the need for further refinement, broader validation, and better integration of predictive models into modern clinical practice. Furthermore, the emergence of ML and artificial intelligence-based approaches, which offer personalized and adaptive predictions, has overshadowed traditional statistical methods [5] and offers a promising alternative for predicting natural conception.

A notable study by Bachelot et al. (2021) introduced a couple-based ML model that combines anthropometric, antioxidative, and metabolic signatures to stratify fertile and infertile couples [8]. This model differs significantly from traditional approaches by treating the couple as a single unit of analysis, rather than evaluating male and female factors independently. Using a dataset of 97 infertile couples with idiopathic infertility and 100 fertile couples, the model achieved an accuracy of 73.8% after refining from 80 variables to 13 features. The most influential predictors included glycemia, abdominal obesity markers, and key antioxidants such as retinol, alpha-carotene, and beta-carotene, which are modifiable through lifestyle interventions​. However, a key limitation of this model is its reliance on laboratory-based variables, which require couples to undergo extensive biochemical and metabolic testing. Predictors such as glycemia and antioxidative markers necessitate access to specialized healthcare facilities, making this approach less accessible for routine primary care settings. Additionally, the model's dependence on hospital-based data highlights a potential barrier for couples who may not have immediate access to such resources. As a result, while the model demonstrates strong predictive capabilities, its practical use is currently constrained to clinical environments with advanced laboratory infrastructure.

In our study, we aimed to develop an ML model to predict the likelihood of natural conception using readily available sociodemographic and health-related data. By analyzing data from 98 couples with confirmed natural pregnancies and 99 couples who were unable to conceive despite regular, unprotected sexual intercourse for one year, we constructed multiple ML models. Among these, the XGB Classifier demonstrated the best performance, achieving an accuracy of 62.5%. However, despite being the best-performing model, metrics such as the ROC-AUC value (0.580) were not as high as desired, reflecting the challenges of predicting natural conception with the current dataset and selected features. Out of the 63 variables collected, 25 were selected as features through the feature selection process. This process significantly reduced noise and improved model interpretability while maintaining its predictive power. Interestingly, the selected variables represented all genders and encompassed a balance of medical, lifestyle, and reproductive factors, emphasizing the couple-based approach of this study. For instance, among the 63 variables, some collected data, such as chemical exposure, secondary sexual characteristics, or family planning history, were retained as key predictors due to their significant influence on fertility. The feature selection process highlighted the critical role of other variables, for example: BMI, age, reproductive history (e.g., regular menstruation or varicocele), and systemic conditions in understanding natural conception. This aligns with existing literature, which frequently emphasizes these factors as key determinants of fertility. Additionally, lifestyle factors such as caffeine consumption and regular exercise emerged as significant predictors, underscoring the importance of modifiable behaviors in fertility outcomes. Conversely, certain variables, despite their clinical relevance, such as a history of thromboembolism or acne, were not selected. This suggests that these features, while important in individual cases, may have limited value in predicting natural conception across a diverse population.

In comparison with existing statistical models, such as the Hunault [1], Bensdorp [2], and dynamic prediction models [3], our study presents several strengths when compared to the notable work by Bachelot et al. [4]. The ML approach that we developed in this study utilizes readily accessible sociodemographic and health-related data from both partners. By leveraging complex interactions between non-invasive predictors, our model provides personalized predictions for distinguishing between couples likely to conceive naturally and those experiencing unexplained infertility. This approach enhances feasibility and cost-effectiveness, making it particularly suitable for primary care settings.

Despite its strengths, our study has certain limitations that should be acknowledged. First, while the model demonstrated promising performance using non-invasive, readily available predictors, the sample size of 98 fertile and 99 infertile couples may limit the generalizability of the results to larger and more diverse populations. Future studies with broader datasets across different geographic and clinical settings are needed to validate the model’s robustness. Second, although the data were not self-administered but were collected face-to-face by physicians using a structured form, the reliance on retrospective participant responses—particularly regarding lifestyle factors and reproductive history—may still introduce recall bias or inaccuracies, potentially impacting the model’s predictive accuracy. Third, our model includes comprehensive sociodemographic and health-related variables but does not incorporate laboratory-based or dynamic clinical data, such as hormonal profiles or metabolic markers, which could further enhance predictive performance. However, such additions would compromise the model’s core strength of being cost-effective and applicable in settings lacking access to specialized diagnostics. Fourth, while we utilized widely accepted ensemble learning algorithms such as Random Forest, LightGBM, and XGBoost, it is important to acknowledge their limitations, particularly when applied to couple-based data. These models are inherently built for independent observations and do not account for the relational structure between partners. Moreover, the randomness embedded in their architecture—such as random feature selection or split point determination—can lead to variability in performance, especially with smaller datasets. Although we attempted to mitigate this through cross-validation and by treating each couple as a unified analytical unit, this approach does not fully capture the complex interdependence between partners. Future research may benefit from applying relational or graph-based models specifically designed for pair-level analysis. Fifth, although we implemented several machine learning algorithms and compared their outcomes using standard performance metrics, we did not apply extensive hyperparameter tuning, advanced ensemble stacking, or post hoc interpretability tools. Our primary aim was to evaluate the feasibility of predicting natural conception with accessible and interpretable ML techniques. Future work should include more technically sophisticated ML pipelines to enhance both performance and insight generation. In addition to these limitations, the model has not been externally validated using an independent dataset, which is essential to confirm its applicability and reproducibility across different populations. Lastly, although feature selection effectively reduced dimensionality and improved interpretability, it may have excluded variables that are clinically important in specific subgroups. Furthermore, the study design does not incorporate longitudinal follow-up, limiting our ability to model dynamic changes in conception probability over time.

Future research should also explore the validation of the model in underrepresented populations—such as individuals from low-resource settings, non-heteronormative couples, and those with limited access to fertility care—to ensure inclusivity and equity. Longitudinal designs that follow couples over extended periods could provide more dynamic insights into the evolving probability of natural conception. Finally, while maintaining the accessibility of the model, future versions might explore the integration of minimally invasive biomarkers or behavioral interaction patterns between partners to further improve predictive capacity. Future studies should also focus on refining the model through larger, externally validated datasets and exploring its integration into public health initiatives, where it could play a pivotal role in early infertility detection and fertility preservation.

The variables incorporated in our study represent fundamental factors influencing couples'chances of natural conception. However, the awareness of these factors may vary significantly depending on the level of reproductive health knowledge within different populations. This highlights the need for proactive measures to improve fertility awareness and accessibility to predictive tools. In the future, integrating ML models into user-friendly platforms, such as mobile applications or web-based tools, could enable couples to assess their likelihood of natural conception even before actively attempting pregnancy. Such tools could serve as a screening test, alerting couples to their potential risk of infertility. Early identification of these risks could prompt timely clinical evaluation and interventions, preventing delays in addressing underlying reproductive health issues. Furthermore, the model could be incorporated into fertility awareness programs as a routine assessment for all couples, enhancing education about reproductive health while promoting informed decision-making. By utilizing non-invasive, easily obtainable predictors, this approach ensures wide applicability across diverse populations and healthcare settings.

While our model offers a non-invasive and potentially cost-effective approach to early fertility assessment, several real-world challenges may affect its clinical implementation. Ethical concerns arise from the possibility of false positives or negatives influencing couples'emotional well-being or delaying appropriate care; thus, AI-assisted predictions should always be interpreted within a clinical context and accompanied by professional counseling. Data privacy is another critical issue, particularly when handling sensitive reproductive health information; robust data protection protocols and secure infrastructure are essential for deployment. In addition, variability in digital literacy among both patients and healthcare providers may limit the accessibility or usability of such tools, especially in low-resource settings. Finally, regulatory frameworks for AI in medicine are still evolving, and standardized guidelines for model validation, approval, and clinical integration will be necessary before such systems can be widely adopted in fertility care.

In conclusion, this study represents the first attempt in the literature to predict natural conception using a machine-learning approach based solely on a combination of sociodemographic and health-related data from both partners. For the machine learning research community, our findings underscore the potential of using structured, non-clinical data in predictive modeling, while highlighting the limitations of standard ensemble methods in capturing relational dynamics. This paves the way for future research involving graph-based or relational ML models specifically tailored to couple-level fertility prediction. For the field of obstetrics and gynecology, the proposed model presents a novel early screening tool that can support timely referral and intervention in primary care settings, particularly in populations lacking access to specialized infertility diagnostics. Future validation of this model on larger, externally verified datasets, along with its integration into mobile or web-based platforms, could significantly enhance its value for reproductive health screening and awareness.

## Supplementary Information

Below is the link to the electronic supplementary material.

Supplementary file1 (DOCX 18 KB)

Supplementary file2 (DOCX 13 KB)

## Data Availability

The data that support the findings of this study are available from the corresponding author upon reasonable request.
