# Supplementary material for: A Novel Machine Learning Model for Predicting Natural Conception Using Non-Laboratory-Based Data
Source: Reprod Sci. 2025 Jul 14;32(8):2644–53. doi: 10.1007/s43032-025-01927-2 (PMC12361258; doi:10.1007/s43032-025-01927-2)
Supplement: Supplementary file 1 — Supplementary file1 (DOCX 18 KB) [file 43032_2025_1927_MOESM1_ESM.docx]

Table 1a. Baseline characteristics of female partners

|  | **Median** | **5–95th**  **percentile** |
| --- | --- | --- |
| Age | 30.0 | 23.0 - 39.0 |
| BMI | 26.4 | 21.6 - 35.8 |
| Age of Menarche | 13.0 | 12.0 - 16.0 |
| Age of Puberty | 13.0 | 11.0 - 16.0 |
| Menstrual Duration (days) | 5.0 | 3.0-7.0 |
|  | **Present (n. %)** | **Absent (n. %)** |
| Smoking | 12 (6.1) | 185 (93.9) |
| Alcohol Consumption | 4 (2.0) | 193 (98.0) |
| Daily Caffeine Use | 81 (41.1) | 116 (58.9) |
| Exercise | 58 (29.4) | 139 (70.6) |
| Depression History | 19 (9.6) | 178 (90.4) |
| Continuous Medication Use | 48 (24.4) | 149 (75.6) |
| Systemic Disease History | 22 (11.2) | 175 (88.8) |
| Thromboembolism History | 1 (0.5) | 196 (99.5) |
| Family History of Thromboembolism | 9 (4.6) | 188 (95.4) |
| Cancer History | 12 (6.1) | 185 (93.9) |
| Chemotherapy/Radiotherapy | 6 (3.0) | 191 (97.0) |
| STD History | 2 (1.0) | 195 (99.0) |
| Genetic Disease History | 11 (5.6) | 186 (94.4) |
| Abdominal Surgery History | 35 (17.8) | 162 (82.2) |
| Uterine Surgery History | 34 (17.3) | 163 (82.7) |
| Congenital Uterine Problems | 8 (4.1) | 189 (95.9) |
| Endometriosis History | 11 (5.6) | 186 (94.4) |
| Hormonal Problem History | 26 (13.2) | 171 (86.8) |
| Acne History | 31 (15.7) | 166 (84.3) |
| Regular Menstruation | 173 (87.8) | 24 (12.2) |
| PCOS History | 42 (21.3) | 155 (78.7) |
| Hirsutism History | 41 (20.8) | 156 (79.2) |
| Vaginismus History | 13 (6.6) | 184 (93.4) |
| Family Planning History | 54 (27.4) | 143 (72.6) |
| Emergency Contraceptive Use | 9 (4.6) | 188 (95.4) |
| Chemical Exposure History | 0 (0) | 197 (100.0) |
| Family Infertility History | 10 (5.1) | 187 (94.9) |
| Family Premature Ovarian Failure | 6 (3.0) | 191 (97.0) |

BMI: Body Mass Index, STD: sexual transmitted disease
