# Supplementary material for: A Novel Machine Learning Model for Predicting Natural Conception Using Non-Laboratory-Based Data
Source: Reprod Sci. 2025 Jul 14;32(8):2644–53. doi: 10.1007/s43032-025-01927-2 (PMC12361258; doi:10.1007/s43032-025-01927-2)
Supplement: Supplementary file 2 — Supplementary file2 (DOCX 13 KB) [file 43032_2025_1927_MOESM2_ESM.docx]

Table 1b. Baseline characteristics of male partners

|  | **Median** | **5–95th**  **percentile** |
| --- | --- | --- |
| Age | 30.0 | 23.0 - 39.0 |
| BMI | 26.4 | 21.6 - 35.8 |
| Puberty Onset Age | 13.0 | 12.0 - 16.0 |
|  | **Present (n. %)** | **Absent (n. %)** |
| Smoking | 104 (52.8) | 93 (47.2) |
| Alcohol Consumption | 59 (29.9) | 138 (70.1) |
| Daily Caffeine Use | 107 (54.3) | 90 (45.7) |
| Exercise | 85 (43.1) | 112 (56.9) |
| Depression History | 9 (4.6) | 188 (95.4) |
| Continuous Medication Use | 26 (13.2) | 171 (86.8) |
| Heat Exposure | 17 (8.6) | 180 (91.4) |
| Systemic Disease History | 15 (7.6) | 182 (92.4) |
| Thromboembolism History | 1 (0.5) | 196 (99.5) |
| Family History of Thromboembolism | 2 (1.0) | 195 (99.0) |
| Cancer History | 9 (4.6) | 188 (95.4) |
| Chemotherapy/Radiotherapy | 4 (2.0) | 193 (98.0) |
| STD History | 1 (0.5) | 196 (99.5) |
| Genetic Disease History | 3 (1.5) | 194 (98.5) |
| Cryptorchidism History | 3 (1.5) | 194 (98.5) |
| Secondary Sex Characteristics | 7 (3.6) | 190 (96.4) |
| Post-Pubertal Mumps History | 6 (3.0) | 191 (97.0) |
| Orchitis History | 0 (0) | 197 (100) |
| Testicular Trauma History | 2 (1.0) | 195 (99.0) |
| Varicocele | 26 (13.2) | 171 (86.8) |
| Varicocelectomy History | 14 (7.1) | 183 (92.9) |
| Testicular Surgery | 16 (8.1) | 181 (91.9) |
| Erectile Dysfunction | 3 (1.5) | 194 (98.5) |
| Premature Ejaculation | 16 (8.1) | 181 (91.9) |
| Chemical Exposure History | 2 (1.0) | 195 (99.0) |
| Family Planning History | 40 (20.3) | 157 (79.7) |
| Family Infertility History | 5 (2.5) | 192 (97.5) |

BMI: Body Mass Index, STD: sexual transmitted disease
